# Interoception and Positive Symptoms in Schizophrenia

**DOI:** 10.3389/fnhum.2016.00379

**Published:** 2016-07-27

**Authors:** Martina Ardizzi, Marianna Ambrosecchia, Livia Buratta, Francesca Ferri, Maurizio Peciccia, Simone Donnari, Claudia Mazzeschi, Vittorio Gallese

**Affiliations:** ^1^Department of Neuroscience, University of ParmaParma, Italy; ^2^Department of Human Science and Education, University of PerugiaPerugia, Italy; ^3^Department of Psychology, University of EssexColchester, UK; ^4^Sementera Onlus AssociationPerugia, Italy; ^5^Institute of Philosophy, School of Advanced Study, University of LondonLondon, UK

**Keywords:** basic self, bodily self, grandiosity, interoception, interoceptive accuracy, positive symptoms, schizophrenia, selfhood

## Abstract

The present study focuses on the multifaceted concept of self-disturbance in schizophrenia, adding knowledge about a not yet investigated aspect, which is the interoceptive accuracy. Starting from the assumption that interoceptive accuracy requires an intact sense of self, which otherwise was proved to be altered in schizophrenia, the aim of the present study was to explore interoceptive accuracy in a group of schizophrenia patients, compared to healthy controls. Furthermore, the possible association between interoceptive accuracy and patients’ positive and negative symptomatology was assessed. To pursue these goals, a group of 23 schizophrenia patients and a group of 23 healthy controls performed a heartbeat perception task. Patients’ symptomatology was assessed by means of the Positive and Negative Syndrome Scale (PANSS). Results demonstrated significantly lower interoceptive accuracy in schizophrenia patients compared to healthy controls. This difference was not accounted for participants’ age, BMI, anxiety levels, and heart rate. Furthermore, patients’ illness severity, attention and pharmacological treatment did not influence their interoceptive accuracy levels. Interestingly, a strong positive relation between interoceptive accuracy and positive symptoms severity, especially Grandiosity, was found. The present results demonstrate for the first time that interoceptive accuracy is altered in schizophrenia. Furthermore, they prove a specific association between interoceptive accuracy and positive symptomatology, suggesting that the symptom Grandiosity might be protective against an altered basic sense of self in patients characterized by higher sensibility to their inner bodily sensations.

## Introduction

“*A circle is the only geometric shape defined by its centre. No chicken and egg about it, the centre came first, the circumference follows. The earth, by definition, has a centre. And only the fool that knows it can go wherever he pleases, knowing the centre will hold him down, stop him flying out of orbit. But when your sense of centre shifts, comes whizzing to the surface, the balance has gone. The balance has gone. The balance my baby has gone.*”

[Sarah Kane, Crave]

These words allow to grasp the core subjective experience of schizophrenia, the loss of the most fundamental selfhood, which is interchangeably named as “minimal self”, “basic self”, “proto-self”, or “ipseity” (Hur et al., 2014). Assuming selfhood as a multi-layered concept, its most primitive, pre-reflective, and immediate layer, which remains when all other levels are stripped away, can be considered as the basic experience of the self (Sass and Parnas, 2003; Fuchs, 2005). In schizophrenia it principally refers to the loss of the basic sense of ownership and agency of one’s own experiences, thoughts, or actions (Jaspers, 1923). Robust empirical evidence indicates that both full-blown psychosis (Peled et al., 2000, 2003; Thakkar et al., 2011; Ferri et al., 2013) and psychosis proneness among non-clinical samples (Morgan et al., 2011; Germine et al., 2013) are associated with a blurred and extremely flexible sense of body ownership, measured by the Rubber Hand Illusion paradigm (RHI, Botvinick and Cohen, 1998). Further studies, although not consistently (for a review see Hur et al., 2014), demonstrated the presence of an altered sense of agency in people suffering from schizophrenia. Indeed, among schizophrenia patients both abnormal over-estimations (Haggard et al., 2003; Voss et al., 2010; Maeda et al., 2012) and under-estimations (Synofzik et al., 2010; Renes et al., 2013) of the causality between one’s own actions and the subsequent external events were found.

Interestingly, specific relations between deficits in basic self experiences and schizophrenia symptomatology have been extensively demonstrated. For instance, Schneiderian first rank symptoms (i.e., thought insertion, thought-broadcasting, somatic passivity, delusional perception) are associated to altered sense of ownership and agency (Fourneret et al., 2001; Jeannerod, 2009; Waters and Badcock, 2010); while weak body ownership is associated to positive schizophrenia symptoms (Peled et al., 2000; Thakkar et al., 2011) and anhedonia negative symptom (Ferri et al., 2014). Furthermore, abnormal over-estimation of individual sense of agency strongly correlates with positive psychotic symptoms severity (Voss et al., 2010) and prevalence (Maeda et al., 2013). Consistently, a significant under-estimation of individual sense of agency appeared to be related with the prevalence of negative psychotic symptoms (Maeda et al., 2013).

Another intriguing aspect of the self experience, which recently gained a lot of attention, is interoception. It is defined as the individual sensitivity to physiological stimuli originating inside of the body (Craig, 2002). Interoception, far from being considered as an unitary concept, can be quantified along the three dimensions of: interoceptive accuracy (i.e., objective performance at behavioral task requiring the detection of visceral sensations); interoceptive sensibility (i.e., explicit self-assessment of subjective interoception abilities by questionnaires) and interoceptive awareness (i.e., metacognitive awareness obtained by confidence-accuracy correspondence) (Garfinkel et al., 2014). The most studied dimension is the interoceptive accuracy, which assumes a fundamental self experience related to the implicit and pre-reflective notion of the self. In fact, the attribution of feelings and sensations to one’s own body presupposes an intact basic sense of self. “*No chicken and egg about it, the centre came first, the circumference follows”*: necessarily, first I implicitly feel myself, then I can attribute internal body sensations to myself. In more empirical terms, a link between interoceptive accuracy and basic self experiences has to be expected. Coherently, low interoceptive accuracy resulted associated to a greater malleability of body sense of ownership among healthy participants (Tsakiris et al., 2011; Tajadura-Jiménez et al., 2012; Suzuki et al., 2013). The strict relation between interoceptive accuracy and basic self experiences finds neuroscientific support in the fact that the most salient inner bodily feelings, contributing to a “cinemascopic representation of the entire body from within”, require the Insular cortex (Craig, 2003) the brain structure also involved in interoceptive processes (Pollatos et al., 2007b; Jarrahi et al., 2015).

The recent growing interest in interoception is justified by two reasons. First, interoceptive accuracy has been demonstrated to play a crucial role in the modulation of numerous aspects of cognitive and affective human life. It influences decision making processes (Werner et al., 2009) as well as the perception and evaluation of emotional stimuli (Pollatos et al., 2007a; Dunn et al., 2010a). Furthermore, it also appears to be involved in the autonomic regulation during social interactions (Ferri et al., 2013) and in individual resilience ability (Haase et al., 2016). Second, interoceptive accuracy appeared to be compromised in several psychiatric disorders, such as anorexia nervosa, major depression, depersonalization-derealization disorders, and anxiety disorders (Pollatos et al., 2008; Furman et al., 2013; Gaudio et al., 2014; Sedeño et al., 2014; Harshaw, 2015).

Remarkably, studies regarding the abilities to perceive one’s own internal bodily signals in schizophrenia are still lacking. This lacuna is particularly important for several reasons. As described above, schizophrenia is characterized by altered experience of the basic sense of self (i.e., body ownership and sense of agency), which has been proved to be related to interoception in healthy participants. Furthermore, the unusual bodily and visceral sensations included in the “coenaesthesic” schizophrenia symptoms (Parnas et al., 2005b; Vollmer-Larsen et al., 2007) (e.g., migrating inner sensations wandering through the body, electric, or thermal feelings, abnormal sense of pulling/pressure or heaviness/emptiness inside of the body, and dysesthetic crises involving the vegetative system) suggest a severe impairment in the patients’ sensitivity to internal bodily signals. Finally, schizophrenia patients show anatomical and functional alterations in the Insular cortex (Kasai et al., 2003; Wylie and Tregellas, 2010; Ebisch et al., 2011, 2013, 2014), which possibly accounts for a large variety of symptoms encompassing affect and pain processing, hallucination (especially visceral hallucinations) (Kathirvel and Mortimer, 2013), self-perception and also visceral abnormal sensations.

All this evidence taken together, in addition to the huge impact of interoceptive accuracy on fundamental cognitive and affective aspects of human life, makes the investigation of interoceptive accuracy in schizophrenia and its potential influence on symptomatology of crucial relevance.

In the present study, interoceptive accuracy was estimated in a group of schizophrenia patients compared to healthy controls. Furthermore, the possible relation between individual intero ceptive accuracy and positive and negative symptomatology was assessed. To these goals, all the participants performed a heartbeat perception task (Schandry, 1981). Furthermore, schizophrenia patients completed a clinical evaluation of their symptomatology by means of the Positive and Negative Syndrome Scale (PANSS) (Kay et al., 1987). The heartbeat perception task was chosen instead of the heartbeat discrimination task – a different technique to assess individual interoceptive accuracy (Brener and Kluvitse, 1988) – because, whereas in the execution of the heartbeat perception task attention is directly focused only on inner bodily signals (i.e., heartbeats), in the heartbeat discrimination task participants are required to integrate internal and external signals (i.e., heartbeats and sounds) to give a synchrony judgment. Given the multisensory integration deficits frequently described in schizophrenia patients (for a review see Tseng et al., 2015), we wanted to rule out any possible confounding effect of these deficits on the assessment of participants’ interoceptive abilities.

Drawing from the prior studies, here briefly revised, we expected to find significantly lower interoceptive accuracy in schizophrenia patients, with respect to healthy controls. Moreover, due to the novelty of the issue a specific relation between interoceptive accuracy and patients’ symptomatology was assessed in an explorative way.

## Materials and Methods

### Participants

Twenty-three schizophrenia patients (SCZ; 17 males, mean age 33.78 years ± 6.33) and 23 healthy controls (HC; 20 males; mean age 31.91 years ± 9.18) were included in the present study. SCZ participants were recruited from outpatient services at Perugia Mental Health Department and diagnosed according to the structured clinical interview for DSM-IV. The mean illness duration was 9.22 ± 3.61 months. Only SCZ participants treated with atypical antipsychotic were included in the study. Due to the large variety of atypical antipsychotics frequently used in the treatment of schizophrenia, an estimation of evidence-based and consistent therapeutic dose equivalence across these medications is needed to directly compare patients’ exposed to different drugs, with different dosages and for different times. For this reason, Chlorpromazine equivalents were calculated following standard practices for antipsychotics (Woods, 2003). Exclusion criteria for all participants comprised significant medical, cardiac or neurological illnesses, substance abuse or dependence in the previous 6 months and mental retardation (IQ < 70). Solely for the HC participants either a personal history of Axis I/II disorders or a history of psychosis in first-degree relatives were considered as exclusion criteria.

All participants filled an anamnestic questionnaire through which their demographic and medical information was obtained. SCZ participants were further evaluated by structured clinical interviews for DSM-IV Axis I (SCID-I) and Axis II (SCID-II) disorders (First et al., 1996, 2012). They were rated for positive and negative symptoms severity using the PANSS for Schizophrenia (including Positive, Negative, and General Psychopathology scales) (Kay et al., 1987) and for their social functioning through the Global Assessment of Functioning scale (GAF) (Hall, 1995). Patients’ intelligence quotient (IQ) was evaluated by means of the Raven Standard Progressive Matrices (SPM) (Raven et al., 1998a,b). Healthy controls’ psychopathological symptoms were evaluated by means of the Symptom Checklist-90-Revised (SCL-90-R) (Derogatis and Savitz, 2000). Finally, to control for individual differences in anxiety at the time of the experiment, all participants filled the State Anxiety Inventory (STAI-I) (Pedrabissi and Santinello, 1989).

See **Table 1** for a detailed description of participants’ information.

**Table 1:** Demographic information about Schizophrenia patients (SCZ) and healthy controls (HC).

	SCZ	HC
n.	23	23
Age (mean ±*SD*)	33.78 ± 6.33	31.91 ± 9.18
Male sex, (*n*° – %)	17–73.91	20–86.96
Right handedness, (n° – %)	20–86.96	21–91.30
Body Mass Index (BMI), Kg/m^2^ (mean ±*SD*)	24.31 ± 2.31	23.27 ± 2.54
Heart Rate (HR), bpm (mean ±*SD*)	86.48 ± 16.21	76.72 ± 15.59
Diagnosis		
Schizophrenia paranoid subtype, (n° – %)	20–86.95	n.a.
Schizoaffective disorder, (n° – %)	3–13	n.a.
Illness duration, year (mean ±*SD*)	9.22 ± 3.61	n.a.
Structured Clinical Interview for DSM-IV Axis II disorders (SCID-II)		
Cluster A, (n° – %)	2–8.69	n.a.
Cluster B, (n° – %)	2–8.69	n.a.
Cluster C, (n° – %)	2–8.69	n.a.
Global Assessment of Functioning Scale (GAF) (mean ±*SD*)	46.70 ± 7.60	n.a.
Symptom Checklist-90-Revised (SCL-90-R) total score (mean ±*SD*)	n.a.	49.44 ± 9.05
Positive and Negative Syndrome Scale for Schizophrenia (PANSS)		
Positive Scale (P) (mean ±*SD*)	22.17 ± 8.16	n.a.
Prevalence of Positive symptoms (n° – %)	10–43.48	n.a.
Delusions (mean ±*SD*)	3.65 ± 1,70	n.a.
Conceptual disorganization (mean ±*SD*)	2.78 ± 1.28	n.a.
Hallucinatory behavior (mean ±*SD*)	2.74 ± 1.54	n.a.
Excitement (mean ±*SD*)	3.00 ± 1.62	n.a.
Grandiosity (mean ±*SD*)	3.09 ± 1.70	n.a.
Suspiciousness/persecution (mean ±*SD*)	3.87 ± 1.58	n.a.
Hostility (mean ±*SD*)	3.04 ± 1.64	n.a.
Negative Scale (N) (mean ±*SD*)	25.74 ± 7.65	n.a.
Prevalence of Negative symptoms (n° – %)	13–56.52	n.a.
General Psychopathology Scale (G) (mean ±*SD*)	53.39 ± 12.42	n.a.
Composite Scale (mean ±*SD*)	-3.57 ± 10.45	n.a.
Total (mean ±*SD*)	101.30 ± 22.38	n.a.
State Anxiety Inventory (STAI-I) (mean ±*SD*)	47.23 ± 14.95	34.43 ± 7.49 ^∗^
Chlorpromazine Equivalent, mg/die (mean ±*SD*)	389.77 ± 762.35	n.a.
Intelligent Quotient (mean ±*SD*)	101.83 ± 13.10	n.a.
Atypical antipsychotic		
Risperidone, (n° – %)	3–13.04	n.a.
Olanzapine, (n° – %)	7–30.43	n.a.
Quetiapine, (n° – %)	1–4.34	n.a.
Ziprasidone, (n° – %)	1–4.34	n.a.
Aripiprazole, (n° – %)	4–17.39	n.a.


*Numbers may not add to total due to missing data or rounding. n.a., not available; ^∗^*p* < 0.05.*

### Procedure

This study was approved by the Bioethics Committee of Perugia University. Written informed consent was obtained from all participants after full explanation of the study procedure, in line with the Declaration of Helsinki 2013.

To avoid potential influences on participants’ heart rate, the assumption of alcohol, caffeine, and tobacco for 2 h prior to the experiment was forbidden to all participants. On arrival, participants filled the self-report questionnaires (i.e., anamnestic questionnaire, SCL-90-R, STAI-I). Before this session, SCZ participants completed the clinical assessment (i.e., DSM-IV interviews, PANSS, GAF, and SPM) at the outpatient service of the Perugia Mental Health Department.

Interoceptive accuracy was measured using the heartbeat perception task (Schandry, 1981; Garfinkel et al., 2014) that has good test–retest reliability (up to 0.81) (Tsakiris et al., 2011) and highly correlates with other detection tasks (Knoll and Hodapp, 1992). Without taking advantage from biological feedback (e.g., by taking their wrist pulse), participants were instructed to silently count their own heartbeats following an audiovisual “start” signal until they received an audiovisual “stop” signal. “Start” and “stop” signals individuated four different time intervals of 100, 45, 35, and 25 s, presented in random order across participants. A brief training session (15 s) was arranged before the actual experiment intervals. At each “stop” signal participants were asked to orally communicate to the experimenter the number of heartbeats counted during the just completed time interval. Both the length of the intervals and the quality of task performance were never revealed to participants.

During the entire procedure, electrocardiogram (ECG) was recorded using three 10 mm Ag/AgCl pre-gelled electrodes (ADInstruments, UK) attached to the participants’ wrists and left ankle following the ordinary Einthoven’s triangle configuration. Before the execution of the heartbeat perception task, participants’ ECG was recorded for 2 additional minutes in a rest condition to collect participants’ baseline heart rate.

Interoceptive accuracy was then calculated, following standard procedure (Schandry, 1981; Garfinkel et al., 2014), as the mean score of four heartbeat perception intervals according to the following formula:

¼Σ(1−(|recorded⁢ heartbeats−counted⁢ heartbeats|)/recorded⁢ heartbeats).

Consequently, interoceptive accuracy values vary between 0 and 1, with higher scores indicating small differences between recorded and counted heartbeats and therefore greater interoceptive accuracy.

## Results

### Between-groups Differences in Age, Body Mass Index, STAI-I Score, and Heart Rate

In order to verify between-groups differences in participants’ age, Body Mass Index (BMI), STAI-I score and heart rate (HR) possibly influencing participants’ interoceptive accuracy (Jones, 1995; Khalsa et al., 2009; Pollatos et al., 2009), four independent sample *t*-tests (two-tailed) were performed comparing SCZ and HC participants. Results demonstrated a significant difference between SCZ and HC for STAI-I score (SCZ: 47.23, SE 3.19; HC: 34.04, SE 1.46; *t*_43_ = 3.814, *p* = 0.001) and HR (SCZ: 86.48 BPM SE 16.21; HC: 76.72 BPM, SE 3.25; *t*_44_ = 2.083, *p* = 0.043). No significant difference was found for age (*t*_44_ = 0.748, *p* = 0.459) and BMI (*t*_44_ = 1.465, *p* = 0.15).

### Between-Groups Difference in Interoceptive Accuracy

**FIGURE 1:**
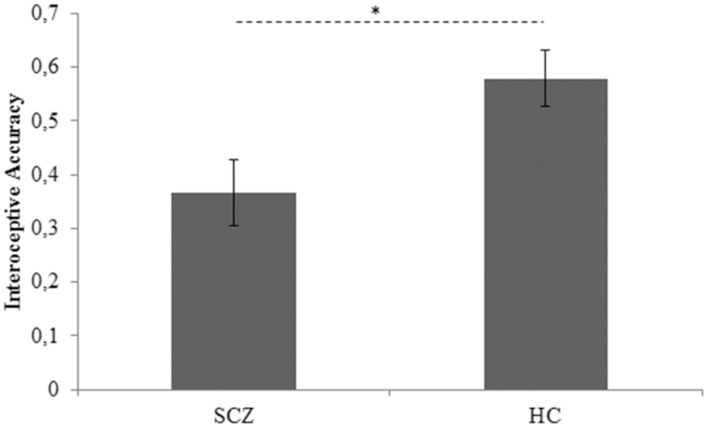
**Interoceptive accuracy marginal means for SCZ and HC participants.** Covariates included in the model were estimated equal to the following values: age = 32.7778; BMI = 23.8401; STAI-I score = 40.6889; HR = 81.4887 SCZ: Schizophrenia patients; HC: Healthy controls. Error bars represent SE. ^∗^*p* < 0.05.

To pursue the first goal of the present study, between-groups difference in interoceptive accuracy, controlling for age, BMI, STAI-I score, and HR, was assessed by an ANCOVA analysis. Group (SCZ, HC) was entered as between-factor, whereas age, BMI, STAI-I score, and HR were included in the model as covariates. The Levene’s test was not significant [*F*_(1,43)_ = 1,558, *p* = 0.219], revealing that the homogeneity of variance assumption was not violated. Results demonstrated that SCZ showed a significant lower interoceptive accuracy than HC (SCZ: 0.366, SE 0.063; HC: 0.579, SE 0.62; *F*_(1,39)_ = 4.355, *p* = 0.043, 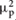 = 0.10) (**Figure 1**). None of the covariates included in the model resulted significant [age: *F*_(1,39)_ = 3.183, *p* = 0.082, 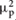 = 0.07; BMI: *F*_(1,39)_ = 0.211, *p* = 0.649, 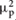 = 0.01; STAI-I score: *F*_(1,39)_ = 0.008, *p* = 0.928, 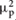 = 0.01; HR: *F*_(1,39)_ = 0.777, *p* = 0.383].

### Impact of SCZ Participants’ Illness Severity, Attention, and Pharmacological Treatment on Interoceptive Accuracy

Illness duration (computed in years from the first psychotic episode), number of hospital admissions and SCZ participants’ score obtained to GAF scale were used as indexes of illness severity. In order to assess the possible influence of illness severity on SCZ participants’ interoceptive accuracy three linear regression analyses were computed including years from the first psychotic event, number of hospital admissions and GAF score as predictors. Similarly, possible attention deficit could interfere with patients’ performance in heartbeat perception task preventing the needed focus on internal bodily signals. For this reason another linear regression analysis was conducted on patients’ interoceptive accuracy using the score obtained at Poor Attention item of PANSS as predictor (G11 score of PANSS). Finally, the possible role of pharmacological treatment (measured by Chlorpromazine equivalents; Woods, 2003) on SCZ participants’ interoceptive accuracy was investigated performing a further linear regression analysis in which Chlorpromazine equivalents was treated as a predictor. Results demonstrated the absence of any significant effect of both illness severity [illness duration: *R*^2^ = 0.166; *F*_(1,17)_ = 3.188; *p* = 0.093; β = 0.408; number of hospital admissions: *R*^2^ = 0.044; *F*_(1,22)_ = 0.977; *p* = 0.334; GAF score: *R*^2^ = 0.156; *F*_(1,22)_ = 3.877; *p* = 0.062]; attention [*R*^2^ = 0.054; *F*_(1,22)_ = 1.204; *p* = 0.285; β = 0.233], and pharmacological treatment [*R*^2^ = 0.037; *F*_(1,15)_ = 0.532; *p* = 0.478; β = 0.191] on SCZ participants’ interoceptive accuracy.

### Relation between Interoceptive Accuracy and Schizophrenia Symptoms

**FIGURE 2:**
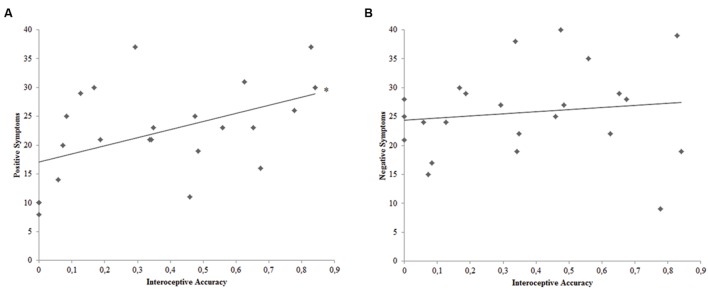
**(A)** Correlation plot of the relation between interoceptive accuracy and PANSS Positive Symptoms Scale score for SCZ participants. **(B)** Correlation plot of the relation between interoceptive accuracy and PANSS Negative Symptoms Scale score for SCZ participants. ^∗^ = Bonferroni corrected *p* < 0.025.

Pearsons’ correlation analyses were conducted to pursue the second goal of this study, thus evaluating the relation between interoceptive accuracy and positive or negative symptomatology among SCZ participants. Bonferroni-corrected (*p* < 0.025) correlation analyses demonstrated a significant relation between SCZ participants’ interoceptive accuracy and the score obtained with the Positive PANSS scale (*r*_23_ = 0.483; *p* = 0.020) (**Figure 2**). Conversely, SCZ participants’ interoceptive accuracy was not related to the score obtained with the Negative PANSS scale (*r*_23_ = 0.132; *p* = 0.547).

**FIGURE 3:**
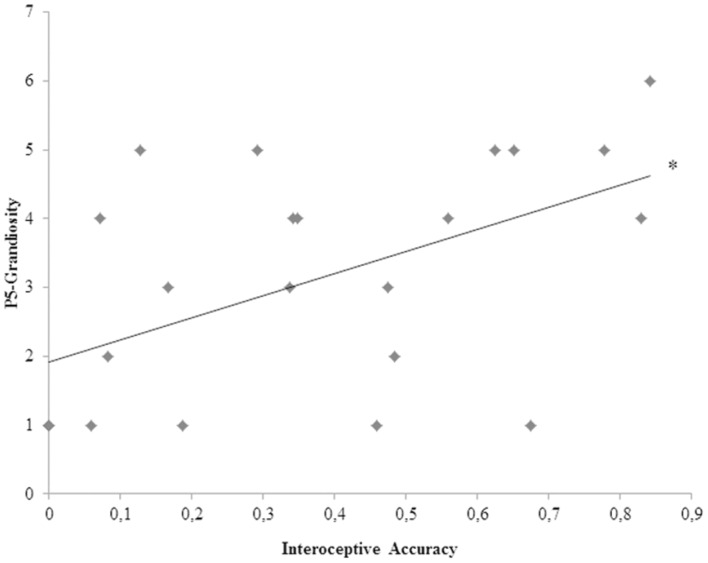
**Correlation plot of the relation between interoceptive accuracy and P5-Grandiosity positive symptom for SCZ participants.** ^∗^*p* value near to the Bonferroni corrected threshold (*p* < 0.007).

To better explore the significant relation between interoceptive accuracy and SCZ participants’ positive symptoms and hence to evaluate which of the seven different positive symptoms showed the strongest relation with SCZ participants’ interoceptive accuracy, Bonferroni-corrected (*p* < 0.007) correlation analyses were calculated for the seven items of the Positive PANSS scale (P1, P2, P3, P4, P5, P6, P7). SCZ participants’ P5-Grandiosity was the only item of the Positive PANSS scale turned out to have a near Bonferroni-corrected significant linear relation with interoceptive accuracy (*r*_23_ = 0.531; *p* = 0.009) (**Figure 3**). See **Table 2** for the Pearsons’ correlation coefficients and *p* values obtained for the all seven items of Positive PANSS scale.

**Table 2:** Pearsons’ correlation coefficients (r) and *p* values (two-tailed) calculated between SCZ participants’ interoceptive accuracy and each item of the PANSS Positive Scale.

		*P*1	*P*2	*P*3	*P*4	*P*5	*P*6	*P*7
Interoceptive Accuracy	*r*	0.437	0.378	0.238	0.410	0.531	0.095	0.384
	*p* (two-tailed)	0.037	0.075	0.274	0.052	0.009^∗^	0.667	0.070
	N.	23	23	23	23	23	23	23


*^∗^*p* value near to the Bonferroni corrected threshold (*p* < 0.007).*

In order to substantiate the relevance of P5-Grandiosity in the relation between interoceptive accuracy and SCZ participants’ positive symptoms, a partial correlation analysis was performed. Partial correlation analysis allows the study of the linear relationship between two variables after excluding the effect of one or more factors.

Thus, if the relation between SCZ participants’ interoceptive accuracy and the score at Positive PANSS scale obtained by the sum of the six items with the exclusion of P5-Grandiosity, results not significant when controlling for the score at P5-Grandiosity, it is reasonable to conclude that P5-Grandiosity is a relevant positive symptom mediating the tested linear relation between interoceptive accuracy and positive symptoms. On the contrary, if the linear relation between interoceptive accuracy and the positive symptoms (with the exclusion of P5-Grandiosity) results significant, also when controlling for P5-Grandiosity, this last symptom can not be considered the principal mediator of the relation of interest. The inclusion of P5-Grandiosity as control variable in this last analysis conducted on the score of Positive PANSS scale obtained by the sum of all items with the exclusion of P5 is necessary to avoid the possible influence of this specific symptom on the other positive symptoms scored in the PANSS positive symptoms scale.

Pearsons’ partial correlation analysis did not show a significant linear relation between interoceptive accuracy and SCZ participants’ score at Positive PANSS scale obtained by the sum of the six items with the exclusion of P5-Grandiosity, when controlling for P5-Grandiosity (*r*_20_ = 0.132; *p* = 0.559). Taken together, these analyses suggested a mediator role of P5-Grandiosity in the relation between SCZ participants’ interoceptive accuracy and positive symptoms.

## Discussion

The present study focused on the basic experience of the self in schizophrenia, more specifically, on a not yet investigated aspect, which is interoceptive accuracy. Starting from the assumption that the effective detection and attribution of inner bodily sensations to oneself requires an intact basic sense of self, the aim of the present study was to explore the individual sensitivity to physiological stimuli originating inside of the body in a group of schizophrenia patients, compared to healthy controls. Furthermore, on the basis of the extended literature connecting altered basic self experiences, such as body ownership and sense of agency, with schizophrenia symptomatology, we also explored possible associations between interoceptive accuracy and positive or negative schizophrenia symptoms.

As expected, results demonstrated significantly lower interoceptive accuracy in schizophrenia patients when compared to healthy controls. This significant difference was not explained by participants’ age, BMI, anxiety levels or HR. Furthermore, patients’ illness severity, attention and pharmacological treatment did not affect their interoceptive accuracy. It is important to note that patients’ attention was assessed by means of the score obtained at the corresponding item in the PANSS (G11 score of PANSS) instead of formal neuropsychological assessment. Future and more focused studies employing a direct evaluation of patients’ attentive abilities are required to totally exclude a possible interfering role of attention on patients’ interoceptive accuracy.

These results show, for the first time, that schizophrenia patients have a reduced sensitivity to their inner bodily signals. This suggests that, besides a feeble body ownership and an iper/ipo-trophic sense of agency, the basic experience of the self, as a body self, in schizophrenia is also characterized by damaged interoceptive accuracy.

Hence, the negative relationship between the malleability of the basic self and the interoceptive accuracy, previously evidenced in healthy participants (Tsakiris et al., 2011; Tajadura-Jiménez et al., 2012; Suzuki et al., 2013), seems to be preserved in schizophrenia patients, where both body ownership (Peled et al., 2000, 2003; Thakkar et al., 2011) and interoceptive accuracy are altered.

Furthermore, considering coenaesthesic symptoms in schizophrenia, described as unusual bodily and visceral sensations (Parnas et al., 2005b; Vollmer-Larsen et al., 2007), the reduced interoception in schizophrenia patients could constitute a previously neglected feature possibly involved in these clinical manifestations.

Several studies have shown that interoception is altered in different psychiatric disorders. Among others, low interoceptive accuracy was established in anorexia nervosa (Pollatos et al., 2008; Gaudio et al., 2014), major depression (Furman et al., 2013; Harshaw, 2015) and depersonalization-derealization disorders (Sedeño et al., 2014). In a different way, interoceptive accuracy was found abnormally higher among people suffering from anxiety disorders than healthy controls (Pollatos et al., 2009; Domschke et al., 2010). Frequently, deficit in interoceptive accuracy has been associated to anatomo-functional alterations of the Insular cortex (see for example, Frank, 2015; Kerr et al., 2015) and to clinical severity (Dunn et al., 2010b; Avery et al., 2014; Forrest et al., 2015; Yoris et al., 2015). In general, the fact that interoception is altered in several psychiatric diseases, suggests an unspecific interaction between mental illnesses and interoceptive accuracy. In the present study, however, we found no general effects of illness severity on patients’ interoceptive accuracy. Rather, there was a linear relation between interoceptive accuracy and only positive symptoms suggesting a specific association between interoceptive abilities and illness qualities of schizophrenia. This specific association was mainly explained by the greater impact of Grandiosity (P5 score of PANSS), with respect to other positive symptoms. Grandiosity positive symptom refers to an “exaggerated self-opinion and unrealistic convictions of superiority, including delusions of extraordinary abilities, wealth, knowledge, fame, power, and moral righteousness”.

A link between interoception and overstated explicit self representation has been established in a large sample of healthy controls (Lyons and Hughes, 2015). The authors demonstrated a positive relation between narcissistic traits and awareness of inner body sensations assessed through a formal questionnaire. In a similar vein, when healthy participants were asked to concentrate on their own mirror image (Ainley et al., 2012), the presentation of self-related words or photograph of themselves (Ainley et al., 2013) increased their interoceptive accuracy.

Overall, it seems that high self-opinion or focused attention on explicit aspects of the self are associated to increased sensitivity to the internal signals of the body. Drawing from this evidence, we speculated that while interoception might contribute to boost the explicit self representation in healthy controls, it might contribute to a pathologically hyperbolic explicit self representation in schizophrenia patients, characterized by a distorted sense of self. Grandiosity and grandiose delusions among schizophrenia patients, as well as narcissism traits in healthy participants, are indeed frequently described as defensive compensations against failures, dissatisfactions with life and traumatic events (Knowles et al., 2011; Lyons and Hughes, 2015). From this point of view, grandiosity and grandiose delusions might be protective also against the altered basic sense of self characterizing schizophrenia patients with higher sensibility to inner bodily sensations. The loss of “*the circumference centre*” might find its compensation by artificially building an explicit over-extended self, particularly among patients who are more in tune with their own internal bodily signals.

In sum, the present results suggest that even if interoceptive accuracy is altered in different psychiatric disorders, in the case of schizophrenia it has a specific association with the clinical profile of patients.

The present work specifically focuses on interoceptive accuracy (i.e., objective performance at behavioral task requiring the detection of visceral sensations), conceived as the most basic dimension of interoception underlying both interoceptive sensibility (i.e., explicit self-assessment of subjective interoception abilities by questionnaires) and interoceptive awareness (i.e., metacognitive awareness obtained by confidence-accuracy correspondence) (Garfinkel and Critchley, 2013; Garfinkel et al., 2014). The three interoceptive dimensions, however, were found to correlate only in people with high interoceptive accuracy (Garfinkel et al., 2014). Thus, despite the fundamental qualification of interoceptive accuracy, conclusions on this dimension cannot be generalized to the other two. Specifically, the fact that schizophrenia patients show lower interoceptive accuracy does not necessarily mean that they also would show low interoceptive sensibility. For example, the Hyperreflexivity tendency, considered one of the complementary aspects of ipseity disorder (Sass and Parnas, 2003), predictive of schizophrenia symptomatology (Sass and Parnas, 2003, 2007; Sass et al., 2013), may be conceptually closer to interoceptive sensibility than to interoceptive accuracy. Hyperreflexivity refers to an exaggerated self-consciousness, a fundamentally non-volitional tendency for focal, objectifying attention directed toward processes and phenomena that would normally be “inhabited” or experienced as part of oneself. In schizophrenia this exaggerated self-reflection becomes automatic, leading to the popping-out of irrelevant background stimuli (Sass and Parnas, 2003, 2007; Sass et al., 2013). As a consequence, it may also result in an exaggerated interoceptive sensibility. Differently, metacognitive deficits, extensively established in schizophrenia (Gumley, 2011; Lysaker et al., 2014) also at the neural level (van der Meer et al., 2010), may be conceptually closer to altered interoceptive awareness, which is indeed defined as the metacognitive awareness of interoceptive accuracy. Further controlled studies are warranted to better clarify possible deficits of the different interoceptive dimensions in schizophrenia, as well as their specific association with different basic experiences of the self. For example, besides low body ownership and altered sense of agency, recent empirical evidence demonstrated that first-episode schizophrenia patients show an absence of the non-conceptual and pre-reflective experience of self underpinned by sensorimotor processes (Ferri et al., 2012). The relations between such processes and interoceptive dimensions have not been investigated yet, neither in healthy individuals nor in schizophrenia patients.

Finally, the here reported direct association between high interoceptive accuracy and positive symptoms can be more deepened by the additional involvement of larger clinical sample and by the formal assessment of patients’ subjective self experiences through specific instruments (e.g., Examination of Anomalous Self Experience; Parnas et al., 2005a). This effort, adding operational depth to blur concepts, may also overcome critics (see for example, Mishara, 2007) frequently raised to schizophrenia phenomenological models.

## Conclusion

The first time we investigated interoceptive accuracy in schizophrenia, shedding light on a new and not yet investigated aspect of basic self experiences. Schizophrenia patients showed a severe loss of the ability to detect internal bodily signals and to attribute them to themselves. Furthermore, interoceptive accuracy was associated to patients’ positive symptomatology, likely fostering a grandiose, and probably defensive, self-opinion.

## Author Contributions

MAr designed the study, collected, analyzed, and interpreted the data, finally she wrote the manuscript. MAm was involved in the study design, data collection and analyses. She also contributed to the drafting of the manuscript. LB was principally engaged in participants recruitment and data collection, furthermore she gave her contribution to results interpretation. FF designed the study, interpreted the data and drafted the manuscript. MP, SD, and CM were involved in participants’ recruitment and data collection, they also took part to the results interpretation. VG designed the study, interpreted the data and drafted the manuscript. All authors approved the final version of the manuscript.

## Conflict of Interest Statement

The authors declare that the research was conducted in the absence of any commercial or financial relationships that could be construed as a potential conflict of interest.
